# Outcomes in Patients With Mild Traumatic Brain Injury Without Acute Intracranial Traumatic Injury

**DOI:** 10.1001/jamanetworkopen.2022.23245

**Published:** 2022-08-17

**Authors:** Debbie Y. Madhok, Robert M. Rodriguez, Jason Barber, Nancy R. Temkin, Amy J. Markowitz, Natalie Kreitzer, Geoffrey T. Manley

**Affiliations:** 1 Department of Emergency Medicine, University of California San Francisco, San Francisco; 2 Department of Neurology, University of California San Francisco, San Francisco; 3 Department of Neurological Surgery, University of Washington, Seattle; 4 Department of Biostatistics, University of Washington, Seattle; 5 Brain and Spinal Injury Center, Zuckerberg San Francisco General Hospital, San Francisco, California; 6 Department of Emergency Medicine, University of Cincinnati, Cincinnati, Ohio; 7 Department of Neurological Surgery, University of California San Francisco, San Francisco

## Abstract

**Importance:**

Traumatic brain injury (TBI) affects millions of people in the US each year. Most patients with TBI seen in emergency departments (EDs) have a Glasgow Coma Scale (GCS) score of 15 and a head computed tomography (CT) scan showing no acute intracranial traumatic injury (negative head CT scan), yet the short-term and long-term functional outcomes of this subset of patients remain unclear.

**Objective:**

To describe the 2-week and 6-month recovery outcomes in a cohort of patients with mild TBI with a GCS score of 15 and a negative head CT scan.

**Design, Setting, and Participants:**

This cohort study analyzed participants who were enrolled from January 1, 2014, to December 31, 2018, in the Transforming Research and Clinical Knowledge in Traumatic Brain Injury (TRACK-TBI) study, a prospective, observational cohort study of patients with TBI that was conducted in EDs of 18 level I trauma centers in urban areas. Of the total 2697 participants in the TRACK-TBI study, 991 had a GCS score of 15 and negative head CT scan and were eligible for inclusion in this analysis. Data were analyzed from September 1, 2021, to May 30, 2022.

**Main Outcomes and Measures:**

The primary outcome was the Glasgow Outcome Scale-Extended (GOS-E) score, which was stratified according to functional recovery (GOS-E score, 8) vs incomplete recovery (GOS-E score, <8), at 2 weeks and 6 months after the injury. The secondary outcome was severity of mild TBI–related symptoms assessed by the Rivermead Post Concussion Symptoms Questionnaire (RPQ) total score.

**Results:**

A total of 991 participants (mean [SD] age, 38.5 [15.8] years; 631 male individuals [64%]) were included. Of these participants, 751 (76%) were followed up at 2 weeks after the injury: 204 (27%) had a GOS-E score of 8 (functional recovery), and 547 (73%) had a GOS-E scores less than 8 (incomplete recovery). Of 659 participants (66%) followed up at 6 months after the injury, 287 (44%) had functional recovery and 372 (56%) had incomplete recovery. Most participants with incomplete recovery reported that they had not returned to baseline or preinjury life (88% [479 of 546]; 95% CI, 85%-90%). Mean RPQ score was 16 (95% CI, 14-18; *P* < .001) points lower at 2 weeks (7 vs 23) and 18 (95% CI, 16-20; *P* < .001) points lower at 6 months (4 vs 22) in participants with a GOS-E score of 8 compared with those with a GOS-E score less than 8.

**Conclusions and Relevance:**

This study found that most participants with a GCS score of 15 and negative head CT scan reported incomplete recovery at 2 weeks and 6 months after their injury. The findings suggest that emergency department clinicians should recommend 2-week follow-up visits for these patients to identify those with incomplete recovery and to facilitate their rehabilitation.

## Key Points

**Question:**

What are the 2-week and 6-month functional outcomes of patients with traumatic brain injury (TBI) who presented in the emergency department with a Glasgow Coma Scale (GCS) score of 15 and without acute intracranial traumatic injury detected on computed tomography (ie, negative head CT scan)?

**Findings:**

In this cohort study of 991 participants with TBI with GCS score of 15 and negative head CT scan, 27% had functional recovery and 73% had incomplete recovery 2 weeks after the injury. At 6 months after the injury, 44% had functional recovery and 56% had incomplete recovery.

**Meaning:**

Findings of this study suggest that emergency department clinicians recommend a 2-week follow-up for patients with TBI, a GCS score of 15, and a negative head CT scan to identify those with incomplete recovery and to facilitate their rehabilitation.

## Teaser

This cohort study evaluates the short-term (2 weeks) and long-term (6 months) functional outcomes in patients with traumatic brain injury with Glasgow Coma Scale score of 15 and no intracranial traumatic injury detected on computed tomography.

## Introduction

Traumatic brain injury (TBI) is traditionally classified as severe, moderate, or mild. The classification is based on the patient’s initial Glasgow Coma Scale (GCS) score. The most common category, mild TBI, encompasses a broad spectrum of presentations, ranging from patients with a GCS score of 15 and without acute intracranial traumatic injury detected on brain computed tomography (CT) scan to those with a GCS score of 13 and substantial brain injury on CT scan. Although all-cause mortality for mild TBI is low, studies have found that patients are at a substantial risk for serious sequelae in the first 6 months after injury.^1,2^ In fact, 21% of patients will experience mental health problems, including posttraumatic stress disorder and depression^3^; cognitive and behavioral impairment; and changes in memory, attention, and motivation, which are associated with loss of work days and unemployment.^4^

The largest group of patients with mild TBI, those with a GCS score of 15 and without acute intracranial injury on CT scan (negative head CT scan), which is sometimes referred to as concussion, is typically managed solely by emergency department (ED) personnel.^5,6,7^ Medical care practitioners often assume that these patients will improve over time with no long-term sequelae, and therefore these clinicians may not provide patients with sufficient psychoeducation and follow-up.^8,9,10,11^ Although more than half of the patients with mild TBI at level I trauma centers experience disability 12 months after the injury,^3^ only 42% receive educational materials at discharge and less than half see a medical practitioner within 3 months of the injury.^12^ Few analyses have been undertaken to map the likely trajectories and ultimate outcomes of these patients, leaving little evidence to guide management decisions.^13,14,15,16^

In the current study, we sought to fill this critical knowledge gap by describing the 2-week and 6-month recovery outcomes in a cohort of patients with mild TBI with a GCS score of 15 and a negative head CT scan. Using data from the prospective, multicenter, longitudinal observational cohort study Transforming Research and Clinical Knowledge in Traumatic Brain Injury (TRACK-TBI), we examined the 2-week and 6-month functional vs incomplete recovery outcomes, as measured with the Glasgow Outcome Scale-Extended (GOS-E) score, and burden of mild TBI–related symptoms, as measured with the Rivermead Post Concussion Symptoms Questionnaire (RPQ) total score.

## Methods

### Study Design and Setting

For this cohort study, we abstracted data from the TRACK-TBI study database, details of which have been previously reported.^3^ Briefly, the TRACK-TBI study enrolled patients with TBI at 18 level I trauma centers in the US from January 1, 2014, to December 31, 2018. All 18 study sites obtained approval from their local institutional review board before study initiation. Patients or their legal representatives provided written informed consent to participate. The present cohort study received approval from the University of California, San Francisco Institutional Review Board. We followed the Strengthening the Reporting of Observational Studies in Epidemiology (STROBE) reporting guideline.

Inclusion criteria for the TRACK-TBI study were (1) age 17 years or older, (2) head trauma within 24 hours of presentation, (3) receipt of a head CT scan as part of clinical care, and (4) reporting or showing evidence of alteration in consciousness or amnesia. Participants met the American Congress of Rehabilitation Medicine definition for TBI, the most widely accepted criteria for TBI, which include any period of loss of consciousness; any loss of memory for events immediately before or after the accident, referred to as posttraumatic amnesia; and any alteration in mental state at the time of the accident, including feeling dazed, disoriented, or confused; or focal neurological deficits that may or may not be transient.^17^ Exclusion criteria were severe, life-threatening illness; incarceration; psychiatric holds or debilitating mental health disorders; pregnancy; and nonsurvivable physical trauma. At 4 of the 18 sites, Spanish-speaking patients were included, but non-English speakers were otherwise excluded.

The presence or absence of intracranial injury on head CT scan was confirmed by a blinded, central, board-certified neuroradiologist. All other demographic data, including race and ethnicity, years of education, and clinical factors, were self-reported by participants. Race and ethnicity were assessed alongside other factors, such as years of education, which have been shown to have associations with outcome after mild traumatic brain injury in previous studies.

### Outcomes

The primary clinical outcome was the GOS-E score at 2 weeks and 6 months after the injury. The GOS-E is the most widely used primary outcome measure in TBI and is recommended by several international bodies, including the National Institutes of Health.^18^ With a scale of 1 to 8 (with 1 indicating death; 4, upper severe disability; 5, lower moderate disability; and 8, full recovery or return to baseline function), the GOS-E assesses recovery in the following domains: dependence in and outside of the home and ability to work or study, shop and travel, participate in social and leisure activities, and resume family relationships and friendships, as well as evaluates symptoms. A GOS-E score of 7 indicates symptoms that interfere with the person returning to their preinjury functioning.^19,20,21^ Although the GOS-E was not designed as an outcome assessment tool in mild TBI, we used GOS-E scores of 8 and less than 8 as comparators because a score of 8 has been shown to be a fair estimate for return to baseline function.^22^ For outcome assessment, research associates, who were trained before the study by a certified neuropsychology coordinator, conducted 2-week and 6-month GOS-E assessments either by phone or during in-person clinical visits with the patient or, if the patient required assistance with activities of daily living, with the caregiver (ie, a GOS-E score of 3 or 4). For this analysis of patients with a GCS score of 15 and a negative head CT scan, we categorized participants according to a binary outcome of functional recovery or return to baseline (GOS-E score of 8) vs incomplete recovery (GOS-E score <8) at 2 weeks and 6 months after the injury.

The secondary outcome was the severity of mild TBI–related symptoms (16 new or worsened symptoms since the injury, including headache; dizziness; nausea; and cognitive, mood, and sleep disturbances) as measured using the RPQ.^23^ With scores ranging from 0 (best) to 64 (worst), the RPQ assesses physical symptoms and severity of symptoms experienced in the past week vs before the injury with a rating of 0 indicating none, 1 indicating no more of a problem, 2 indicating mild problem, 3 indicating moderate problem, and 4 indicating severe problem. By convention, a rating of 1 was recorded as 0 for analysis, and a total score compiling all symptoms was used for analysis.

### Statistical Analysis

Differences in patient and injury characteristics among participants with functional recovery and those with incomplete recovery by 6 months are summarized in Table 1 as risk ratios (RR) for incomplete recovery. All statistics in Table 1 were weighted using inverse probability weighting to help account for bias owing to dropout. Corresponding unweighted statistics for functional and incomplete recovery are provided in eTable 1 in Supplement 1. Differences among participants with and without 6-month GOS-E assessments are also reported in eTable 1 in Supplement 1, and these differences were tested for statistical significance using Mann-Whitney tests for continuous or ordinal variables and Fisher exact tests for categorical variables. Two-sided *P* < .05 were considered to be significant, and no adjustment was made for multiple comparisons. Both unweighted and weighted tests were performed to ascertain the effectiveness of the weighting in balancing the 2 cohorts on these characteristics. Table 2 reports the rate of impairment within each GOS-E domain for each level of the total GOS-E score (1-5, 6, 7, and 8) at both 2 weeks and 6 months, as well as the median RPQ score. Cross-tabulation summaries between the 2-week and 6-month assessments are shown in Table 3 for GOS-E scores and Table 4 for RPQ scores.

**Table 1.** Baseline Characteristics of Participants and 6-Month Outcomes^a^

Variable	Overall, No. (%)^b^	6-mo GOS-E outcome				
		Recovery, No. (%)		Unfavorability		Unknown GOS-E score, No. (%)
		Incomplete: GOS-E score of 1-7	Functional: GOS-E score of 8	Risk, %	RR for incomplete recovery (95% CI)^c^	
All participants	991	372	287	56		332
Age, y						
Mean (SD)	38.5 (15.8)	38.4 (15.0)	38.3 (17.0)		1.00 (0.92-1.09) per ≥10 y	37.8 (15.1)
Median (IQR)	34 (24-50)	37 (25-51)	32 (25-52)			34 (25-48)
<30	402 (41)	146 (39)	127 (44)	53	1 [Reference]	132 (40)
30-64	521 (53)	207 (56)	135 (47)	60	1.13 (0.99-1.30)	181 (55)
≥65	68 (7)	19 (5)	24 (8)	44	0.83 (0.58-1.18)	19 (6)
Sex						
Male	631 (64)	208 (56)	204 (71)	51	1 [Reference]	227 (68)
Female	360 (36)	164 (44)	83 (29)	66	1.31 (1.15-1.49)	105 (32)
Race and ethnicity^d^						
African American or Black	215 (22)	89 (24)	58 (21)	60	1.12 (0.95-1.32)	61 (19)
Asian	30 (3)	9 (3)	12 (4)	44	0.81 (0.50-1.33)	5 (2)
Hispanic or Latinx	185 (19)	71 (19)	40 (14)	64	1.19 (1.01-1.41)	101 (31)
Native American, Hawaiian, or Pacific Islander	19 (2)	7 (2)	7 (2)	51	0.94 (0.56-1.58)	3 (1)
Non-Hispanic or Latinx White	535 (54)	195 (53)	167 (59)	54	1 [Reference]	157 (48)
Years of education						
Mean (SD)	13.4 (2.7)	13.2 (2.7)	14.0 (2.7)		0.54 (0.32-0.92) per ≥4 y	12.4 (2.8)
Median (IQR)	12 (12-16)	12 (12-15)	14 (12-16)			12 (11-14)
No college degree	696 (73)	288 (78)	172 (62)	63	1.44 (1.20-1.72)	258 (85)
College degree	260 (27)	80 (22)	104 (38)	43	1 [Reference]	46 (15)
Psychiatric history						
No	761 (77)	254 (68)	243 (85)	51	1 [Reference]	278 (84)
Yes	229 (23)	119 (32)	44 (15)	73	1.43 (1.26-1.62)	53 (16)
Employment status						
Full-time	584 (61)	222 (61)	176 (64)	56	1 [Reference]	194 (65)
Part-time	122 (13)	49 (13)	31 (11)	61	1.10 (0.90-1.33)	31 (10)
Occasional, special, or unemployed	88 (9)	36 (10)	23 (8)	62	1.10 (0.88-1.37)	32 (11)
Retired, disabled, or not working	99 (10)	38 (10)	28 (10)	57	1.03 (0.82-1.29)	28 (9)
Student or other^e^	58 (6)	22 (6)	18 (6)	55	0.99 (0.74-1.33)	15 (5)
Insurance status						
Private insurance or Medicare coverage	603 (63)	217 (59)	197 (71)	52	1 [Reference]	175 (58)
Medicaid or other coverage^e^	150 (16)	77 (21)	26 (9)	75	1.43 (1.24-1.65)	48 (16)
No insurance	198 (21)	72 (20)	56 (20)	56	1.07 (0.89-1.28)	80 (26)
Cause of injury						
MVC occupant	410 (41)	166 (45)	99 (35)	63	1 [Reference]	161 (48)
MCC	82 (8)	33 (9)	22 (8)	59	0.95 (0.75-1.20)	30 (9)
MVC, cyclist or pedestrian	132 (13)	42 (11)	51 (18)	45	0.72 (0.57-0.92)	28 (8)
Fall	200 (20)	66 (18)	71 (25)	48	0.77 (0.63-0.94)	59 (18)
Assault	55 (6)	21 (6)	11 (4)	65	1.05 (0.80-1.37)	24 (7)
Other or unknown^e^	112 (11)	45 (12)	33 (11)	58	0.93 (0.76-1.15)	30 (9)
Nature of cause of injury						
Intentional	44 (4)	16 (4)	8 (3)	66	1 [Reference]	21 (6)
Unintentional	931 (95)	351 (95)	271 (96)	56	0.86 (0.64-1.16)	306 (93)
Undetermined	9 (1)	3 (1)	3 (1)	52	0.79 (0.34-1.85)	3 (1)
Anticoagulant use						
No	949 (98)	360 (98)	279 (99)	56	1 [Reference]	307 (98)
Yes	18 (2)	9 (2)	4 (1)	71	1.25 (0.87-1.80)	6 (2)
Loss of consciousness						
None	95 (13)	28 (10)	31 (15)	48	1 [Reference]	32 (14)
<30 min	589 (82)	234 (84)	168 (82)	58	1.22 (0.92-1.61)	188 (80)
≥30 min	34 (5)	16 (6)	5 (2)	78	1.63 (1.15-2.32)	14 (6)
Posttraumatic amnesia						
None	166 (25)	56 (22)	60 (30)	48	1 [Reference]	50 (23)
<30 min	328 (49)	132 (51)	89 (45)	60	1.24 (1.00-1.54)	105 (48)
≥30 min	182 (27)	73 (28)	49 (25)	60	1.24 (0.98-1.58)	62 (29)
Prehospital hypotension						
No	857 (98)	332 (98)	235 (97)	59	1 [Reference]	300 (99)
Yes	19 (2)	7 (2)	8 (3)	45	0.77 (0.43-1.35)	3 (1)
Prehospital hypoxia						
No	851 (98)	334 (99)	234 (96)	59	1 [Reference]	292 (97)
Yes	20 (2)	4 (1)	9 (4)	28	0.48 (0.20-1.15)	8 (3)
Urine toxicology screen result						
Negative	161 (64)	52 (60)	46 (69)	53	1 [Reference]	65 (64)
Positive	89 (36)	34 (40)	21 (31)	62	1.17 (0.89-1.55)	37 (36)
Blood alcohol concentration						
Mean (SD)	40 (91)	31 (81)	43 (97)		0.97 (0.90-1.05), per ≥40 mg/dL	46 (95)
<80	517 (84)	211 (87)	135 (83)	61	1 [Reference]	182 (81)
≥80	102 (16)	32 (13)	27 (17)	55	0.89 (0.70-1.14)	44 (19)
ED hypotension						
No	980 (99)	367 (99)	285 (99)	56	1 [Reference]	328 (99)
Yes	11 (1)	5 (1)	2 (1)	70	1.25 (0.78-2.00)	4 (1)
ED hypoxia						
No	968 (98)	365 (98)	279 (97)	57	1 [Reference]	325 (98)
Yes	23 (2)	7 (2)	8 (3)	50	0.88 (0.52-1.46)	7 (2)

Abbreviations: ED, emergency department; GOS-E, Glasgow Outcome Scale-Extended; MCC, motorcycle collision; MVC, motor vehicle collision; RR, risk ratio.

^a^ Inverse probability weighting was used to help account for bias owing to participants who were not followed for the 6-month GOS-E assessment; hence, the GOS-E counts within a covariate category may not sum exactly to the overall count.

^b^ Unknown values: race and ethnicity (n = 7); years of education (n = 35); psychiatric history (n = 1); employment status (n = 40); insurance status (n = 40); nature of cause of injury (n = 7); anticoagulant use (n = 24); loss of consciousness (n = 273); posttraumatic amnesia (n = 315); prehospital hypotension (n = 115); prehospital hypoxia (n = 120); urine toxicology screen result (n = 741); blood alcohol concentration (n = 372).

^c^ RRs for age, years of education, and BAC estimated by negative binomial regression.

^d^ Race and ethnicity were self-reported by participants.

^e^ Other status was not available.

**Table 2.** Postinjury GOS-E Scores With Corresponding GOS-E Domain Impairment and RPQ Score^a^

GOS-E score	Participants No. (%)	Percentage of participants impaired per GOS-E domain (95% CI)							RPQ score, median (IQR)	
		Home^b^	Shop^b^	Travel^b^	Work^b^	Social^b^	Family and friends^b^	Return to baseline or preinjury life^b^	2 wk	6 mo
2 wk after injury										
1-5	220 (29)	20 (15-26)	19 (14-25)	20 (15-26)	92 (88-96)	75 (68-80)	53 (46-60)	84 (79-89)	31 (17-42)	19 (8-34)
6	159 (21)	0 (0-2)	0 (0-2)	0 (0-2)	76 (68-83)	57 (49-65)	36 (28-44)	87 (81-92)	21 (10-34)	10 (2-28)
7	168 (22)	0 (0-2)	0 (0-2)	0 (0-2)	0 (0-2)	13 (8-19)	16 (10-22)	93 (87-96)	12 (6-21)	6 (0-16)
8	204 (27)	0 (0-2)	0 (0-2)	0 (0-2)	0 (0-2)	0 (0-2)	0 (0-2)	0 (0-2)	4 (0-10)	0 (0-8)
No. unknown	240	NA	NA	NA	NA	NA	NA	NA	NA	NA
6 mo after injury										
1-5	59 (9)	8 (3-19)	10 (3-20)	9 (3-20)	74 (59-85)	57 (43-70)	78 (65-88)	87 (75-94)	34 (19-44)	35 (22-46)
6	130 (20)	0 (0-3)	0 (0-3)	0 (0-3)	60 (51-69)	38 (30-47)	70 (62-78)	85 (78-91)	31 (15-43)	27 (16-38)
7	183 (28)	0 (0-2)	0 (0-2)	0 (0-2)	0 (0-2)	8 (4-13)	23 (17-29)	88 (82-92)	21 (9-30)	11 (4-19)
8	287 (44)	0 (0-1)	0 (0-1)	0 (0-1)	0 (0-1)	0 (0-1)	0 (0-1)	0 (0-1)	6 (2-14)	0 (0-4)
No. unknown	332	NA	NA	NA	NA	NA	NA	NA	NA	NA

Abbreviations: GOS-E, Glasgow Outcome Scale-Extended; NA, not applicable; RPQ, Rivermead Post Concussion Symptoms Questionnaire.

^a^ Inverse probability weighting was used to help account for bias owing to participants who were not followed for each outcome.

^b^ Definitions of GOS-E domains: Home represents inability to look after self at home; shop, inability to shop; travel, inability to travel; work, inability to work or study; social, inability to participate in social and leisure activities outside the home; family, disruption in family and friend relationships; and return, inability to return to preinjury life.

**Table 3.** Cross-Tabulation Comparing 2-Week With 6-Month GOS-E Outcomes^a^

2-wk GOS-E score	6-mo GOS-E score							
	1	3	4	5	6	7	8	Unknown
1	1	NA	NA	NA	NA	NA	NA	NA
3	NA	1	NA	3	6	6	2	NA
4	NA	NA	1	5	11	7	6	3
5	NA	NA	1	17	44	42	33	5
6	1	NA	2	10	33	41	44	31
7	NA	NA	NA	4	22	48	67	28
8	NA	NA	1	7	9	33	125	26
Unknown	1	NA	NA	7	6	8	8	29

Abbreviations: GOS-E, Glasgow Outcome Scale-Extended; NA, not applicable.

^a^ Inverse probability weighting was used to help account for bias owing to participants who were not followed for each outcome.

**Table 4.** Cross-Tabulation Comparing 2-Week With 6-Month RPQ Outcomes^a^

2-wk RPQ score	6-mo RPQ score						
	0-9	10-19	20-29	30-39	40-49	≥50	Unknown
0-9	230	23	7	NA	2	NA	49
10-19	80	32	20	5	4	2	37
20-29	31	29	18	11	7	1	21
30-39	21	18	19	16	8		19
40-49	9	8	7	16	16	7	11
**≥**50	3	1	2	5	8	8	6
Unknown	14	7	5	6	1	2	136

Abbreviations: NA, not applicable; RPQ, Rivermead Post Concussion Symptoms Questionnaire.

^a^ Inverse probability weighting was used to help account for bias owing to participants who were not followed for each outcome.

The summaries in Table 1 to Table 4 use inverse propensity weighting to help account for any potential selection bias owing to incomplete follow-up, defined as a missing GOS-E or RPQ score at the relevant time point. With this method, all participants are given more (or less) weight in each analysis according to how much they resemble (or differ from) participants who were not followed up. All variables exactly as they appear in Table 1 were used to inform the propensity modeling. Boosted logistic regression models were constructed, using 5000 iterations and a shrinkage factor of 0.01 with a Kolmogorov-Smirnov estimator to assess balance and considering all possible 2-way and 3-way covariate interactions, to estimate the propensity of each participant in the analysis to have functional recovery at follow-up. These propensities were then converted to statistical weights by inverting and then rescaling them such that the mean weight of the sample remained equal to 1.

Statistical testing was carried out using SPSS, version 26 (IBM SPSS Statistics). Boosted regression models were constructed using the Toolkit for Weighting and Analysis of Nonequivalent Groups applet (Rand Corporation).^24^ Data were analyzed from September 1, 2021, to May 30, 2022.

## Results

Of the total 2697 participants in TRACK-TBI study, 1706 (63%) were excluded from the present analysis because of age, GCS score less than 15, CT scan findings positive for acute intracranial traumatic injury, or unknown GCS or CT findings. Included in the analysis were 991 patients (37%) who had a GCS score of 15 and no evidence of intracranial injury on CT scan. Of these participants, 751 (76%) completed 2-week follow-up GOS-E assessments and 659 (66%) completed the 6-month follow-up GOS-E assessments. The flowchart of included participants is presented in Figure.

**Figure.**
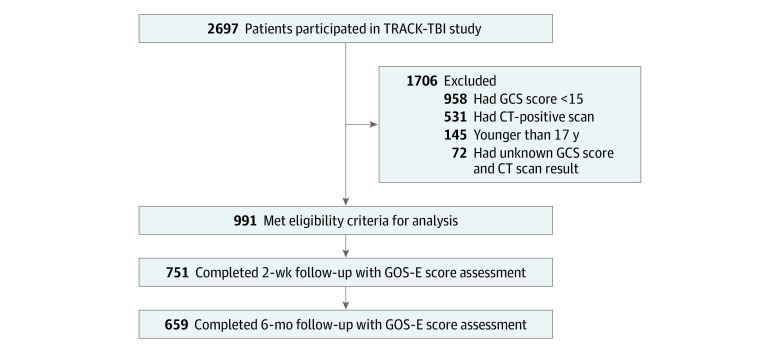
Flowchart of Participants CT indicates computed tomography; GCS, Glasgow Coma Scale; GOS-E, Glasgow Outcome Scale-Extended; and TRACK-TBI, Transforming Research and Clinical Knowledge in Traumatic Brain Injury.

Participants had a mean (SD) age of 38.5 (15.8) years; 631 were men (64%) and 360 were women (36%). More than 50% of participants had non-Hispanic or Latinx White race and ethnicity (535 [54%]). The median (IQR) number of years of education was 12 (12-16) years; 229 participants (23%) had a psychiatric history, and 198 participants (21%) had no insurance. In terms of presentation, 589 participants (82%) reported loss of consciousness lasting less than 30 minutes, whereas 95 (13%) did not experience loss of consciousness. Moreover, 328 participants (49%) had posttraumatic amnesia lasting less than 30 minutes, 182 (27%) had posttraumatic amnesia lasting at least 30 minutes, and 166 (25%) did not have posttraumatic amnesia. Participant characteristics, stratified according to 6-month GOS-E outcomes, are presented in Table 1.

### Primary and Secondary Outcomes

Based on bivariate associations (Table 1), at the 6-month outcome, participants with incomplete recovery (GOS-E score <8) reported fewer median (IQR) years of education compared with those with functional recovery (GOS-E score of 8) (12 [12-15] years vs 14 [12-16] years), and those without a college degree were more likely to have incomplete recovery than those with a college degree (RR, 1.44; 95% CI, 1.20-1.72). Female participants were more likely than male participants to have incomplete recovery (RR, 1.31; 95% CI, 1.15-1.49). Individuals with a psychiatric history (eg, depression, anxiety, posttraumatic stress disorder, and schizophrenia) were more likely to have incomplete recovery than those without a psychiatric history (RR, 1.43; 95% CI, 1.26-1.62). Participants with Medicaid insurance had higher rates of incomplete recovery compared with participants with private insurance or Medicare coverage (RR, 1.43; 95% CI, 1.24-1.65).

Table 2 shows inverse propensity–weighted summary statistics for each GOS-E subgroup (scores of 1-5, 6, 7, and 8) at 2 weeks and 6 months after the injury, including impairment rates for each corresponding GOS-E domain, and mean or median RPQ scores at both assessments. At 2 weeks, 204 participants (27%) had a GOS-E score of 8 (functional recovery), whereas 547 (73%) had GOS-E scores less than 8 (incomplete recovery). Of 168 participants with a GOS-E score of 7 at 2 weeks, 13% (22; 95% CI, 8%-19%) were unable to resume social activities outside the home, 16% (27; 95% CI, 10%-22%) experienced a disruption in family relationships and friendships, and 93% (156; 95% CI, 87%-96%) were unable to return to baseline or preinjury life. Furthermore, 159 participants (21%) had a GOS-E score of 6 at 2 weeks and reported disruptions in work (121 [76%; 95% CI, 68%-83%]), social activities (91 [57%; 95% CI, 49%-65%]), family relationships and friendships (57 [36%; 95% CI, 28%-44%]), and inability to return to preinjury life (138 [87%; 95% CI, 81%-92%]). More than a quarter of the cohort (220 [29%]) had a GOS-E score of 1 to 5.

At 6 months after the injury (Table 2), 287 participants (44%) had experienced functional recovery, whereas 372 (56%) had incomplete recovery. Of 183 participants with a GOS-E score of 7 at 6 months, 8% (15; 95% CI, 4%-13%) had difficulty returning to social activities outside the home, 23% (42; 95% CI, 17%-29%) had disruptions in family relationships and friendships, and 88% (161; 95% CI, 82%-92%) were unable to return to preinjury life. Of 130 participants with a GOS-E score of 6 at 6 months, 60% (78; 95% CI, 51%-69%) reported disruptions in work, 38% (49; 95% CI, 30%-47%) had difficulty returning to social activities outside the home, 70% (91; 95% CI, 62%-78%) had disruptions in family relationships and friendships, and 85% (111; 95% CI, 78%-91%) were unable to return to preinjury life. Fifty-nine participants (9%) had a GOS-E score of 1 to 5.

Participants with a GOS-E score of 8 at 2 weeks after the injury were more likely to have maintained functional recovery at 6 months after the injury (71% [125 of 175]; 95% CI, 64%-78%) compared with participants with a GOS-E score less than 8 and were more likely to achieve functional recovery at 6 months (33% [152 of 458]; 95% CI, 29%-38%). However, only 48% of participants (67 of 141; 95% CI, 39%-56%) with a GOS-E score of 7 at 2 weeks had functional recovery by 6 months, whereas 34% of participants (44 of 131; 95% CI, 26%-42%) with a GOS-E score of 6 at 2 weeks had functional recovery at 6 months. For those with a GOS-E score of 1 to 5 at 2 weeks, 22% (41 of 186; 95% CI, 16%-29%) had achieved functional recovery by 6 months.

With regard to the secondary outcome of mild TBI–related symptoms (Table 2), participants with functional recovery at 2 weeks after the injury had a median (IQR) RPQ score of 4 (0-10) at 2 weeks and 0 (0-8) at 6 months after the injury. Those with a GOS-E score of 7 at 2 weeks had a median (IQR) RPQ score of 12 (6-21) at 2 weeks and 6 (0-16) at 6 months. Participants with a GOS-E score of 6 at 2 weeks had a median (IQR) RPQ score of 21 (10-34) at 2 weeks and 10 (2-28) at 6 months. Those with a GOS-E score of 1 to 5 at 2 weeks had a median (IQR) RPQ score of 31 (17-42) at 2 weeks and 19 (8-34) at 6 months. Most participants with incomplete recovery reported that they had not returned to baseline or preinjury life (88% [479 of 546]; 95% CI, 85%-90%). Mean RPQ score was 16 (95% CI, 14-18; P < .001) points lower at 2 weeks (7 vs 23) and 18 (95% CI, 16-20; *P* < .001) points lower at 6 months (4 vs 22) in participants with a GOS-E score of 8 compared with those with a GOS-E score less than 8.

Temporal associations of 2-week and 6-month RPQ scores were similar to those of 2-week and 6-month GOS-E scores (Table 3 and Table 4). Of the 262 participants with a 2-week RPQ score between 0 and 9, 88% (230; 95% CI, 83%-91%) also had an RPQ score between 0 and 9 at 6 months. Of the 172 participants with an RPQ score of at least 30 at 2 weeks, 19% (33; 95% CI, 14%-26%) had an RPQ score of 0 to 9 at 6 months, whereas 49% (84; 95% CI, 41%-57%) had an RPQ score of at least 30 at 6 months.

## Discussion

In this cohort study of patients who presented to level I trauma centers in the US with symptoms suggestive of brain trauma, only 27% of those with a GCS score of 15 and without intracranial injury detected on head CT scan were back to preinjury baseline at 2 weeks and only 44% reported a functional recovery at 6 months after the injury. Participants without functional recovery at 6 months described difficulty with returning to social activities outside the home, disruptions in family relationships and friendships, and an inability to return to baseline or preinjury life. Participants who had not fully recovered at 2 weeks after the injury had concerns in similar domains. The findings at 2 weeks were associated with those at 6 months: participants with functional recovery (GOS-E score <8) at 2 weeks were more likely to have maintained functional recovery at 6 months, and those with incomplete recovery (GOS-E score of 8) at 2 weeks were more likely to have incomplete recovery at 6 months. This pattern was also observed when comparing 2-week with 6-month RPQ scores: patients with complete recovery had lower mean RPQ scores than participants with incomplete recovery.

The population in this study had a high rate of preinjury psychiatric comorbidities, and participants with psychiatric comorbidities were more likely to have incomplete recovery than those without psychiatric comorbidities. This finding aligns with results in previous work, which found that patients with mild TBI and concomitant psychiatric disorders had worse recovery than those without preinjury psychiatric comorbidities.^25,26^ In addition, a previous study found that patients who presented to the ED had a higher rate of psychiatric comorbidities than the general population,^27^ making the findings in the present study generalizable to the ED patient population.

These findings highlight the importance of ED clinicians being aware of the risk of incomplete recovery for patients with a mild TBI (ie, GCS score of 15 and negative head CT scan) and providing accurate education and timely referral information before ED discharge. Specifically, ED clinicians should recommend prompt follow-up care, ideally within 2 weeks, because several evidence-based interventions may help to mitigate potentially adverse outcomes, which the data from this study suggest can be estimated at this early time point. Incomplete recovery may even be anticipated while in the ED on the basis of demographic and clinical factors that are apparent or collected in the ED, such as sex, psychiatric history, and educational level. These characteristics emphasize the need to explore the implications of social determinants of health for TBI outcomes.

In one study, patients with TBI who were seen within 1 week of injury and instructed on coping strategies showed improvements in sleep, anxiety, paranoia, and hostility.^28^ Another study found that both cognitive behavioral therapy and cognitive rehabilitation were beneficial for emotional distress, anxiety, and depression after TBI.^29^ In addition, 76% to 97% of postacute care patients with TBI exhibited varying degrees of lack of awareness of their injury, which contributed to inappropriate long-term goals and employment expectations, which can exacerbate perceived dysfunction and emotional distress.^30,31^ Helping patients adjust to and become aware of changes in cognitive function after TBI is critical for their rehabilitation, and interventions that are focused on self-awareness strategies during functional daily living tasks or instrumental activities of daily living have been shown to be associated with improved self-regulation and functional performance in a small pilot study of patients with severe TBI.^32^

Most of the nearly 3 million new cases of TBI in the US each year are classified as mild TBI.^5,6,33^ Better systems of care for patients with TBI must be established, with a focus on bridging care from the ED. The myriad shortcomings in care coordination for TBI may be exacerbated in geographic locales with less access to specialty clinics.^34,35^ These shortcomings are also magnified in racial and ethnic groups who are disproportionately affected by TBI^36,37^ and less likely to receive follow-up care.^38,39,40^ Clinicians in the ED are on the front line of improving the pathways to appropriate follow-up. Some of these pathways include providing specific follow-up appointments before ED discharge, incorporating ED navigator systems into primary care and TBI clinic follow-up, and disseminating tailored educational materials and discharge instructions in multiple formats (eg, paper, verbal, and video) and different languages.

### Limitations

This study has several limitations. Six-month follow-up assessments were missing in nearly a third of study participants, which is consistent with other TBI studies.^41^ This unassessed cohort was composed of substantially more participants of Hispanic ethnicity who had fewer years of education, a lower rate of psychiatric history, less insurance, and were injured more frequently in motor vehicle collisions and assaults. Inverse propensity weighting successfully mitigated all of these imbalances but the education variable (eTables 1-3 in Supplement 1). In comparing against the unweighted complete case analysis, there were only minimal deviations among measures of outcome, with no discernable pattern in either direction. Thus, although the exclusion of the unassessed participants did not appear to change this particular analysis, important latent variables may still exist. Given that the TRACK-TBI study sites were all level I trauma centers in urban areas, the findings of the present work may not apply to patients in other care settings. Because the outcome assessments took place at level I trauma centers and within the framework of a study, the inequity of TBI systems of care may be less apparent, given that patients were given follow-up appointments and incentives to participate in follow-up for outcome measurements. Exclusion of some non-English speakers may have further limited the generalization to broad populations. Furthermore, biases such as social desirability bias and hindsight bias may have affected participants’ reporting of symptoms given that outcomes were based solely on self-report.

## Conclusions

In this cohort study, most patients with TBI with GCS score of 15 and negative head CT scan treated at level I trauma center EDs experienced incomplete recovery and mild TBI–related symptoms at 2 weeks and 6 months after the injury. Given that most patients with TBI are managed solely by ED clinicians, understanding their outcomes is particularly relevant for ED care and disposition planning. The findings of this study suggest that ED clinicians should recommend 2-week follow-up visits for these patients to identify those with incomplete recovery and to facilitate their rehabilitation.

## Notes

Supplement 1. **eTable 1.** Differences in Baseline Characteristics for Participants With and Without Follow-up for 6-Month GOSE, and With Favorable and Unfavorable Outcome (Unweighted) **eTable 2.** GOSE Scores at 2 Weeks and 6 Months Post-Injury With Corresponding GOSE Domain Impairment and RPQ (Unweighted) **eTable 3.** Cross Tabulation Comparing 2 Week and 6 Month Proportion of GOSE and RPQ Outcomes (Unweighted)

Supplement 2.
Nonauthor Collaborators
