# Supplementary material for: Outcomes in Patients With Mild Traumatic Brain Injury Without Acute Intracranial Traumatic Injury
Source: JAMA Netw Open. 2022 Aug 17;5(8):e2223245. doi: 10.1001/jamanetworkopen.2022.23245 (PMC9386538; doi:10.1001/jamanetworkopen.2022.23245)
Supplement: Supplement 1. — eTable 1. Differences in Baseline Characteristics for Participants With and Without Follow-up for 6-Month GOSE, and With Favorable and Unfavorable Outcome (Unweighted) eTable 2. GOSE Scores at 2 Weeks and 6 Months Post-Injury With Corresponding GOSE Domain Impairment and RPQ (Unweighted) eTable 3. Cross Tabulation Comparing 2 Week and 6 Month Proportion of GOSE and RPQ Outcomes (Unweighted) [file jamanetwopen-e2223245-s001.pdf]

Supplemental Online Content

Madhok DY, Rodriguez R, Barber J, et al; TRACK-TBI Investigators. Outcomes in patients with mild traumatic brain injury without acute intracranial traumatic injury. *JAMA Netw Open*. 2022;5(8):e2223245. doi:10.1001/jamanetworkopen.2022.23245

eTable 1. Differences in Baseline Characteristics for Participants With and Without Follow-up for 6-Month GOSE, and With Favorable and Unfavorable Outcome (Unweighted)

eTable 2. GOSE Scores at 2 Weeks and 6 Months Post-Injury With Corresponding GOSE Domain Impairment and RPQ (Unweighted)

eTable 3. Cross Tabulation Comparing 2 Week and 6 Month Proportion of GOSE and RPQ Outcomes (Unweighted)

This supplemental material has been provided by the authors to give readers additional information about their work.

© 2022 Madhok DY et al. *JAMA Network Open.*

eTable 1. Differences in Baseline Characteristics for Participants With and Without Follow-up for 6-Month GOSE, and With Favorable and Unfavorable Outcome (Unweighted)

	**Overall** [1] Column Percents	**Followed for 6-Month GOSE** Column Percents				**6-Month GOSE** Column Percents	
		**No**	**Yes**	**P-Value**		**Incomplete Recovery** (1-7)	**Functional Recovery** (8)
				**Unwt.**	**Wgt.**		
**Participants**	**991**	**332**	**659**			**367**	**292**
**Age**							
Mean (SD)	38.5 (15.8)	37.8 (15.1)	38.8 (16.1)	.507	.889	39.0 (15.2)	38.6 (17.2)
Median (IQR)	34 (24-50)	34 (25-48)	35 (24-51)			38 (24-50)	31 (24-51)
<30	402 (41%)	132 (40%)	270 (41%)	.518	.823	140 (38%)	130 (45%)
30-64	521 (53%)	181 (55%)	340 (52%)			205 (56%)	135 (46%)
65+	68 (7%)	19 (6%)	49 (7%)			22 (6%)	27 (9%)
**Sex**							
Male	631 (64%)	227 (68%)	404 (61%)	.030	.530	203 (55%)	201 (69%)
Female	360 (36%)	105 (32%)	255 (39%)			164 (45%)	91 (31%)
**Race/Ethnicity**							
African American / Black	215 (22%)	61 (19%)	154 (23%)	<.001	.370	93 (25%)	61 (21%)
Asian	30 (3%)	5 (2%)	25 (4%)			11 (3%)	14 (5%)
Hispanic/Latinx	185 (19%)	101 (31%)	84 (13%)			54 (15%)	30 (10%)
Native American/Hawaiian/PI	19 (2%)	3 (1%)	16 (2%)			8 (2%)	8 (3%)
White, Non-Hispanic/Latinx	535 (54%)	157 (48%)	378 (58%)			200 (55%)	178 (61%)
**Years of Education**							
Mean (SD)	13.4 (2.7)	12.4 (2.8)	13.8 (2.5)	<.001	.001	13.4 (2.4)	14.3 (2.7)
Median (IQR)	12 (12-16)	12 (11-14)	13 (12-16)			12 (12-16)	14 (12-16)
No college degree	696 (73%)	258 (85%)	438 (67%)	<.001	.063	272 (75%)	166 (58%)
College degree	260 (27%)	46 (15%)	214 (33%)			93 (25%)	121 (42%)
**Psychiatric History**							
No	761 (77%)	278 (84%)	483 (73%)	<.001	.177	239 (65%)	244 (84%)
Yes	229 (23%)	53 (16%)	176 (27%)			128 (35%)	48 (16%)
**Employment Status**							

© 2022 Madhok DY et al. *JAMA Network Open.*

Full-Time	584 (61%)	194 (65%)	390 (60%)	.284	.782	212 (58%)	178 (62%)
Part-Time	122 (13%)	31 (10%)	91 (14%)			56 (15%)	35 (12%)
Occasional/Special/Unemployed	88 (9%)	32 (11%)	56 (9%)			33 (9%)	23 (8%)
Retired/Disabled/Not Working	99 (10%)	28 (9%)	71 (11%)			40 (11%)	31 (11%)
Student/Other	58 (6%)	15 (5%)	43 (7%)			23 (6%)	20 (7%)
**Insurance**							
Insured/Medicare	603 (63%)	175 (58%)	428 (66%)	<.001	.200	219 (60%)	209 (73%)
Medicaid/Other	150 (16%)	48 (16%)	102 (16%)			77 (21%)	25 (9%)
Uninsured	198 (21%)	80 (26%)	118 (18%)			66 (18%)	52 (18%)
**Cause of Injury**							
MVC Occupant	410 (41%)	161 (48%)	249 (38%)	<.001	.756	154 (42%)	95 (33%)
MCC	82 (8%)	30 (9%)	52 (8%)			31 (8%)	21 (7%)
MVC (cyclist or pedestrian)	132 (13%)	28 (8%)	104 (16%)			46 (13%)	58 (20%)
Fall	200 (20%)	59 (18%)	141 (21%)			68 (19%)	73 (25%)
Assault	55 (6%)	24 (7%)	31 (5%)			20 (5%)	11 (4%)
Other/Unknown	112 (11%)	30 (9%)	82 (12%)			48 (13%)	34 (12%)
**Nature of Cause**							
Intentional	44 (4%)	21 (6%)	23 (4%)	.142	.814	15 (4%)	8 (3%)
Unintentional	931 (95%)	306 (93%)	625 (96%)			347 (95%)	278 (96%)
Undetermined	9 (1%)	3 (1%)	6 (1%)			3 (1%)	3 (1%)
**Anticoagulant Use**							
No	949 (98%)	307 (98%)	642 (98%)	1.000	.353	356 (98%)	286 (99%)
Yes	18 (2%)	6 (2%)	12 (2%)			8 (2%)	4 (1%)
**Loss of Consciousness**							
None	95 (13%)	32 (14%)	63 (13%)	.750	.489	31 (11%)	32 (15%)
<30 min	589 (82%)	188 (80%)	401 (83%)			227 (83%)	174 (83%)
30+ min	34 (5%)	14 (6%)	20 (4%)			16 (6%)	4 (2%)
**Post-Traumatic Amnesia**							
None	166 (25%)	50 (23%)	116 (25%)	.431	.633	55 (22%)	61 (30%)
<30 min	328 (49%)	105 (48%)	223 (49%)			129 (51%)	94 (46%)
30+ min	182 (27%)	62 (29%)	120 (26%)			71 (28%)	49 (24%)
**Pre-Hospital Hypotension**							
No	857 (98%)	300 (99%)	557 (97%)	.092	.481	323 (98%)	234 (96%)
Yes	19 (2%)	3 (1%)	16 (3%)			7 (2%)	9 (4%)

© 2022 Madhok DY et al. *JAMA Network Open.*

**Pre-Hospital Hypoxia**							
No	851 (98%)	292 (97%)	559 (98%)	.637	.647	325 (99%)	234 (96%)
Yes	20 (2%)	8 (3%)	12 (2%)			3 (1%)	9 (4%)
**Urine Tox Screen**							
Negative	161 (64%)	65 (64%)	96 (65%)	.893	.674	50 (61%)	46 (70%)
Positive	89 (36%)	37 (36%)	52 (35%)			32 (39%)	20 (30%)
**Blood Alcohol Concentration**							
Mean (SD)	40 (91)	46 (95)	36 (89)	.432	.886	29 (79)	45 (102)
<80	517 (84%)	182 (81%)	335 (85%)	.144	.905	203 (87%)	132 (83%)
≥80	102 (16%)	44 (19%)	58 (15%)			30 (13%)	28 (18%)
**ED Hypotension**							
No	980 (99%)	328 (99%)	652 (99%)	1.000	.760	362 (99%)	290 (99%)
Yes	11 (1%)	4 (1%)	7 (1%)			5 (1%)	2 (1%)
**ED Hypoxia**							
No	968 (98%)	325 (98%)	643 (98%)	.827	.482	359 (98%)	284 (97%)
Yes	23 (2%)	7 (2%)	16 (2%)			8 (2%)	8 (3%)

1 Unknown values: race/ethnicity (N=7); education (N=35); psychiatric history (N=1); employment status (N=40); insurance (N=40); nature of cause (N=7); anticoagulant use (N=24); loss of consciousness (N=273); post-traumatic amnesia (N=315); pre-hospital hypotension (N=115); pre-hospital hypoxia (N=120); urine tox screen (N=741); blood alcohol concentration (N=372)

2 Inverse propensity weighting was used to try to adjust for any bias due to the unassessed outcomes. P-values are reported for both the unweighted and weighted analyses showing that the propensity model was able to balance the two cohorts on all key variables except education. Only the results of the 6mo GOSE propensity modelling are shown here, and the modelling of the other outcomes yielded similar success.

Note: GOSE, Glasgow Outcome Scale Extended; CT, computed tomography; TBI, traumatic brain injury; GCS, Glasgow Coma Scale; Unk, unknown; MVC, motor vehicle collision; MCC, motorcycle collision; IQR, inter-quartile range; ED, Emergency Department; PI, Pacific Islander; Tox, toxicology; Unwt, unweighted; Wgt, weighted

© 2022 Madhok DY et al. *JAMA Network Open.*

eTable 2. GOSE Scores at 2 Weeks and 6 Months Post-Injury With Corresponding GOSE Domain Impairment and RPQ (Unweighted)

GOSE	N (%)	Percent Impaired on Each GOSE Domain [95% CI]							Rivermead Score Median (IQR)	
		Home	Shop	Travel	Work	Social	Family	Return	2wk	6mo
**2wk**										
**1-5**	213 (28%)	20% [15, 26]	20% [15, 26]	21% [16, 27]	92% [87, 95]	74% [68, 80]	54% [47, 60]	84% [78, 89]	31 (17-43)	17.5 (8-35)
**6**	161 (21%)	0% [0, 2]	0% [0, 2]	0% [0, 2]	77% [69, 84]	58% [50, 65]	35% [28, 43]	88% [81, 92]	21.5 (10-34)	9 (2-28)
**7**	169 (23%)	0% [0, 2]	0% [0, 2]	0% [0, 2]	0% [0, 2]	13% [8, 19]	16% [11, 22]	92% [87, 96]	12 (6-21)	5.5 (0-15)
**8**	208 (28%)	0% [0, 2]	0% [0, 2]	0% [0, 2]	0% [0, 2]	0% [0, 2]	0% [0, 2]	0% [0, 2]	4 (0-10)	0 (0-8)
Unk.	240									
**6mo**										
**1-5**	61 (9%)	8% [3, 18]	10% [4, 20]	8% [3, 18]	75% [60, 86]	57% [44, 70]	79% [66, 88]	87% [76, 94]	34 (20-44)	35 (22-46)
**6**	128 (19%)	0% [0, 3]	0% [0, 3]	0% [0, 3]	63% [53, 72]	41% [32, 50]	68% [59, 76]	86% [79, 91]	31 (16-43)	28 (16-38)
**7**	178 (27%)	0% [0, 2]	0% [0, 2]	0% [0, 2]	0% [0, 2]	7% [4, 11]	22% [16, 29]	88% [83, 93]	18 (8-30)	11 (4-19)
**8**	292 (44%)	0% [0, 1]	0% [0, 1]	0% [0, 1]	0% [0, 1]	0% [0, 1]	0% [0, 1]	0% [0, 1]	6 (2-14)	0 (0-4)
Unk.	332									

Note: GOSE, Glasgow Outcome Scale Extended; RPQ, Rivermead Post Concussion Symptoms Questionnaire. IQR = interquartile range. Home = unable to look after themselves at home; Shop = unable to shop; Travel = unable to travel; Work = unable to work/study; Social = unable to participate in social and leisure activities outside the home; Family = disruption in family and friend relationships; Return = failure to return to normal life; Unk, unknown.

© 2022 Madhok DY et al. *JAMA Network Open.*

eTable 3. Cross Tabulation Comparing 2 Week and 6 Month Proportion of GOSE and RPQ Outcomes (Unweighted)

GOSE 2wk	GOSE 6mo							
	**1**	**3**	**4**	**5**	**6**	**7**	**8**	**Unk.**
**1**	1							
**3**		1		3	6	5	2	3
**4**			1	5	11	7	6	5
**5**			1	17	42	33	33	31
**6**	1		1	10	33	40	48	28
**7**				4	21	49	69	26
**8**			1	7	9	36	126	29
**Unk.**	1			7	6	8	8	210

RPQ 2wk	RPQ 6mo						
	**0-9**	**10-19**	**20-29**	**30-39**	**40-49**	**50+**	**Unk.**
**0-9**	231	24	6		2		49
**10-19**	84	30	20	5	4	2	37
**20-29**	31	29	14	12	8	1	21
**30-39**	21	17	19	15	8		19
**40-49**	9	8	7	17	16	7	11
**50+**	3	1	2	6	8	10	6
**Unk.**	14	7	5	6	1	2	136

Note: GOSE, Glasgow Outcome Scale Extended; Unk, unknown; RPQ, Rivermead Post Concussion Symptoms Questionnaire

© 2022 Madhok DY et al. *JAMA Network Open.*
