# Supplementary material for: Outcomes in Patients With Mild Traumatic Brain Injury Without Acute Intracranial Traumatic Injury
Source: JAMA Netw Open. 2022 Aug 17;5(8):e2223245. doi: 10.1001/jamanetworkopen.2022.23245 (PMC9386538; doi:10.1001/jamanetworkopen.2022.23245)
Supplement: Supplement 2. — Nonauthor Collaborators [file jamanetwopen-e2223245-s002.pdf]

Supplemental Online Content: Nonauthor Collaborators
*First name, last name, and suffix (if applicable) are required and will appear in PubMed.

*Group Name(s): The TRACK-TBI Investigators							
***First Name and Middle Initial(s)**	***Last Name**	***Suffix (eg, Jr, III)**	Academic Degrees	Institution	Location (city, state/province, country)	Role or Contribution, eg, chair, principal investigator	Group (if more than 1 Group listed in the byline) and/or Subgroup (eg, Steering Committee)
Neeraj	Badjatia		MD	University of Maryland			
Ann-Christine	Duhaime		MD	MassGeneral Hospital for Children			
V Ramana	Feeser		MD	Virginia Commonwealth University			
Adam R.	Ferguson		PhD	University of California, San [illegible]			
Raquel	Gardner		MD	University of California, San			
Etienne	Gaudette		MD	University of Toronto			
Shankar	Gopinath		MD	Baylor College of Medicine			
Ramesh	Grandhi		MD, MS	University of Utah			
Ruchira	Jha		MD, MSc	Barrow Neurological Institute			
C. Dirk	Keene		MD, PhD	University of Washington			
Christine	Mac Donald		PhD	University Washington			
Christopher	Madden		MD	UT Southwestern			
Michael	McCrea		PhD	Medical College of Wisconsin			
Randall	Merchant		PhD	Virginia Commonwealth University			
Lindsay	Nelson		PhD	Medical College of Wisconsin			
Laura B.	Ngwenya		MD, PhD	University of Cincinnati			
David	Okonkwo		MD, PhD	University of Pittsburgh			
Claudia	Robertson		MD	Baylor College of Medicine			
David	Schnyer		PhD	UT Austin			
Sabrina R.	Taylor		PhD	University of California, San			
Mary	Vassar		RN, MS	University of California, San			
John K.	Yue		MD	University of California, San			
